# Snake venom rhodocytin induces plasma extravasation via toxin-mediated interactions between platelets and mast cells

**DOI:** 10.1038/s41598-019-52449-2

**Published:** 2019-11-04

**Authors:** Yuki Nakamura, Tomoyuki Sasaki, Chihiro Mochizuki, Kayoko Ishimaru, Schuichi Koizumi, Hideyuki Shinmori, Katsue Suzuki-Inoue, Atsuhito Nakao

**Affiliations:** 1 Department of Immunology, University of Yamanashi, 1110 Shimokato, Chuo, Yamanashi 409-3898 Japan; 2 Department of Clinical and Laboratory Medicine, Faculty of Medicine, University of Yamanashi, 1110 Shimokato, Chuo, Yamanashi 409-3898 Japan; 3 Research Center for Gold Chemistry, Graduate School of Urban Environmental Sciences, Tokyo Metropolitan University, 1-1 Minami-osawa, Hachioji, Tokyo 192-0397 Japan; 4 Department of Pharmacology, Faculty of Medicine, University of Yamanashi, 1110 Shimokato, Chuo, Yamanashi 409-3898 Japan; 5 Synthetic Biology Group, Department of Biotechnology, Faculty of Life and Environmental Science, University of Yamanashi, 4-4-37, Takeda, Kofu 400-8510 Japan; 6 Atopy Research Center, Juntendo University School of Medicine, 2-1-1 Hongo, Bunkyo-ku, Tokyo 113-8421 Japan

**Keywords:** Immunochemistry, Innate immunity

## Abstract

Venomous snakebites can induce local tissue damage, including necrosis of soft tissues, haemorrhage, blistering and local swelling associated with plasma extravasation, which can lead to lethal complications such as hypovolemic shock. However, the details of the underlying mechanisms remain unknown. In this study, we showed that intradermal treatment of mice with venom rhodocytin from the Malayan viper *Calloselasma rhodostoma* induced plasma extravasation, dependent on C-type lectin-like receptor 2 (CLEC-2) on platelets. Rhodocytin-induced plasma extravasation also relied on mast cells and histamine. *In vitro* co-culture of rhodocytin-activated platelets with mast cells induced histamine release from mast cells in an ATP/P2X7-dependent manner. Consistent with this, blockade or deficiency of P2X7 in mast cells suppressed rhodocytin-induced plasma extravasation in the skin. Together, these findings indicate that rhodocytin induces plasma extravasation by triggering platelet activation via CLEC-2, followed by activation of mast cells and histamine release via the ATP/P2X7 pathway. These results reveal a previously unrecognized mechanism by which snake venom increases vascular permeability via complex venom toxin–mediated interactions between platelets and mast cells.

## Introduction

The World Health Organization classifies snakebite as a neglected disease with unmet clinical needs^1^. Venomous snakes can be found on almost every continent, and up to 5 million snakebites occur each year, resulting in about 2.5 million poisonings and 20,000–125,000 deaths^1^. The local common reactions in bitten limb following venomous snakebites are immediate pain, necrosis of soft tissues and extending tender local swelling associated with plasma extravasation, which in some cases (e.g., severe envenoming) leads to hypovolemic shock with multiple organ failure^2,3^. Various substances in venoms, mostly proteins and polypeptides, as well as non-specific tissue injury and inflammation induced by snakebites, are thought to be responsible for their clinical manifestations. However, the precise mechanisms by which venomous snakebites induce plasma extravasation remain unknown.

C-type lectin-like receptor-2 (CLEC-2) was identified as a platelet receptor for rhodocytin (also called aggretin), a snake venom obtained from the Malayan pit viper *Calloselasma rhodostoma*, which is classified as a snake venom C-type lectin known to induce haemorrhage and coagulopathy, having a basic heterodimeric structure with two subunits, α (136 amino acids) and β (123 amino acids)^4–8^ and is a major cause of venomous snakebite morbidity in Southeast Asia^9,10^. In humans, CLEC-2 is highly expressed on platelets/megakaryocytes, and at lower levels in liver^5,6^. Rhodocytin/CLEC-2 interactions activate platelets via protein kinase signaling pathways and induces strong aggregation of platelets, which contributes to the pathology of the venom toxin^5,6^. Subsequently, podoplanin, present in lymphatic endothelial cells, renal podocytes, and several cancer cells, was identified as an endogenous ligand for CLEC-2^11,12^. Interestingly, CLEC-2 expressed on platelets has many functions beyond hemostasis and thrombosis^13–19^. For instance, the interaction of CLEC-2 on platelets with podoplanin on lymphatic endothelial cells is essential for the normal separation of blood/lymphatic vessels during mouse development^13^.

Because CLEC-2 recognizes *C*. *rhodostoma* venom rhodocytin, we hypothesized that CLEC-2 expressed on platelets might play a role in the innate response to snake venom, including plasma extravasation. To test this hypothesis, we administered intradermal injections of rhodocytin into mice and examined the effects of the rhodocytin–CLEC-2 interaction on plasma extravasation in the skin. The results revealed a previously unrecognized mechanism by which snake venom affects vascular permeability in the skin via venom toxin–mediated interactions between platelets and mast cells.

## Results

### Rhodocytin induces plasma extravasation in the skin, dependent upon CLEC-2 expressed on platelets

**Figure 1:**
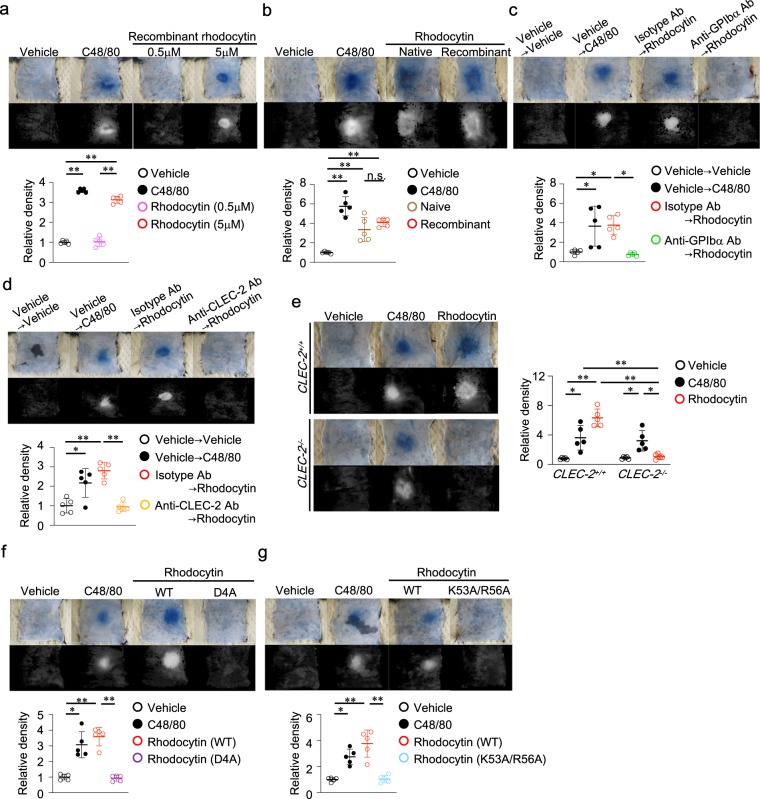
Rhodocytin induces plasma extravasation in the skin, dependent upon CLEC-2 expressed on platelets. (**a**) Representative images of compound 48/80 (C48/80) (10 μg/20 μl i.d.)– or LPS-free recombinant rhodocytin (0.5 or 5 μmol/L/20 μl i.d.)–induced plasma extravasation in wild-type mice (color), and digitized images used for density value evaluations (black and white) (upper panels). Quantitative analysis of the images in the left panel (lower panel). Values represent means ± SD. One-way ANOVA with Bonferroni’s test: *p < 0.05, **p < 0.01 (n = 5). (**b**) Representative images of compound 48/80 (C48/80) (10 μg/20 μl i.d.)- or native (5 μmol/L/20 μl i.d.) or recombinant rhodocytin (5 μmol/L/20 μl i.d.)–induced plasma extravasation in wild-type mice (color), and digitized images used for density value evaluations (black and white) (upper panels). Quantitative analysis of the images in the left panel (lower panel). Values represent means ± SD. One-way ANOVA with Bonferroni’s test: *p < 0.05, **p < 0.01 (n = 5). (**c**,**d**) Representative images of C48/80 (10 μg/20 μl i.d.)- or rhodocytin (5 μmol/L/20 μl i.d.)-induced plasma extravasation in platelet-depleted (**c**) or platelet-selective CLEC-2–depleted (**d**) mice, and digitized images used for density value evaluations (upper panels). Quantitative analysis of the images in the left panels (lower panels). Values represent means ± SD. One-way ANOVA with Bonferroni’s test: *p < 0.05, **p < 0.01 (n = 5). (**e**) Representative images of C48/80 (10 μg/20 μl i.d.)- or rhodocytin (5 μmol/L/20 μl i.d.)-induced plasma extravasation in CLEC-2–deficient irradiated chimeric mice (CLEC-2^−/−^) or control chimeric mice (CLEC-2^+/+^), and digitized images used for density value evaluations (left panels). Quantitative analysis of the images in the left panels (right panel). Values represent means ± SD. One-way ANOVA with Bonferroni’s test: *p < 0.05, **p < 0.01 (n = 5). (**f**,**g**) Representative images of plasma extravasation induced by wild-type or mutated rhodocytins [D4A (**f**) or K53A/R56A (**g**)] (5 μmol/L/20 μl or 10 μmol/L/20 μl i.d.) in wild-type mice, and digitized images used for density value evaluations (upper panels). Quantitative analysis of the images in the left panels (lower panels). Values represent means ± SD. One-way ANOVA with Bonferroni’s test: *p < 0.05, **p < 0.01 (n = 5). (**a**–**g**) Similar results were obtained from at least two independent experiments.

We first investigated whether intradermal (i.d.) injection of rhodocytin would induce plasma extravasation in the skin. Plasma extravasation was visualized 30 minutes after intravenous injection of Evans blue dye followed by i.d. injection of rhodocytin, based on the blue staining of the injection sites on the reverse side of the skin. These staining sites were digitalized using a high-resolution color camera and used for quantitative image analysis as described previously^20^. Intradermal injection of 5 μM LPS-free recombinant rhodocytin^21^ (hereafter, we used this recombinant rhodocytin in all experiments) significantly induced plasma extravasation in wild-type mice (Fig. 1a), as did 5 μM native rhodocytin (Fig. 1b). The effects of rhodocytin were similar to those of i.d. injection of the direct mast cell activator compound 48/80^22^.

To determine whether platelets or platelet-expressed CLEC-2 is required for rhodocytin-induced plasma extravasation in the skin, we compared the effects of i.d. injection of rhodocytin among wild-type mice, platelet-depleted mice (Supplementary Fig. 1a,b), and platelet-selective CLEC-2–deficient mice (Supplementary Fig. 1c,d). Importantly, we observed little plasma extravasation in platelet-depleted or platelet-selective CLEC-2–deficient mice in comparison with control mice (Fig. 1c,d). Consistent with this, mice selectively deficient for CLEC-2 in hematopoietic cells also exhibited little plasma extravasation in the skin following i.d. injection of rhodocytin (Fig. 1e).

Rhodocytin is a tetramer consisting of two α and two β chains: each disulfide-linked dimer consists of an α and a β chain, and two such dimers form a non–disulfide-linked tetramer^23,24^. We recently developed two alanine-substitution mutants in the α- or β-subunit of rhodocytin, αD4AβWT (D4A) or αWTβK53A/R56A (K53A/R56A); the former cannot bind to CLEC-2, whereas the latter binds to, but does not cross-link, CLEC-2, and consequently does not deliver its signal^21^. In contrast to wild-type rhodocytin, neither mutant induced plasma extravasation (Fig. 1f,g). These results suggest that induction of plasma extravasation by rhodocytin is dependent on the rhodocytin/CLEC-2 interaction on platelets, rather than to simple mechanical tissue (vessel) damage at the injection sites.

### Rhodocytin-induced plasma extravasation in the skin requires mast cells and histamine

To understand the overall process by which the rhodocytin/CLEC-2 interaction on platelets induces plasma extravasation, we investigated whether mast cells, as well as their mediator histamine, play a role in rhodocytin-induced plasma extravasation in mouse skin.

**Figure 2:**
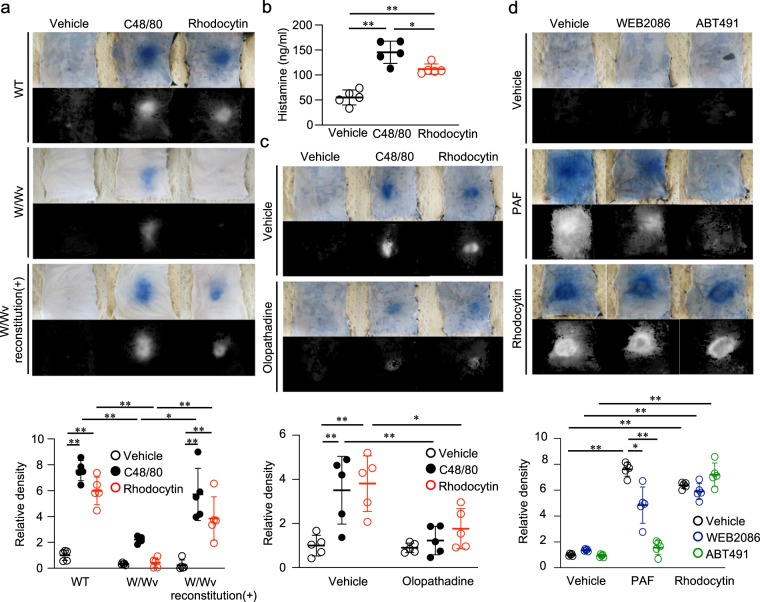
Rhodocytin-induced plasma extravasation in the skin requires mast cells and histamine. (**a**) Representative images of C48/80 (10 μg/20 μl i.d.)- or rhodocytin (5 μmol/L/20 μl i.d.)-induced plasma extravasation in wild-type mice, mast cell–deficient W/Wv mice, or mast cell–deficient W/Wv mice reconstituted with subcutaneous injections of wild-type BMMCs (color), and digitized images used for density value evaluations (black and white) (upper panels). Quantitative analysis of the images in the upper panels (lower panel). Values represent means ± SD. One-way ANOVA with Bonferroni’s test: *p < 0.05, **p < 0.01 (n = 5). (**b**) Plasma samples were collected 10 minutes after i.d. injection of wild-type mice with C48/80 (10 μg/40 μl) or rhodocytin (5 μmol/L/40 μl). Blood levels of histamine were measured with the histamine EIA kit. Values represent means ± SD. One-way ANOVA with Bonferroni’s test: *p < 0.05, **p < 0.01 (n = 5). (**c**) Representative images of C48/80 (10 μg/20 μl i.d.)- or rhodocytin (5 μmol/L/20 μl i.d.)-induced plasma extravasation in wild-type mice pretreated with or without the histamine H1 receptor antagonist olopatadine (10 mg/kg p.o.), and digitized images used for density value evaluations (upper panels). Quantitative analysis of the images in the upper panels (lower panel). Values represent means ± SD. One-way ANOVA with Bonferroni’s test: *p < 0.05, **p < 0.01 (n = 5). (**d**) Representative images of PAF (10 μmol/L/20 μl i.d.)- or rhodocytin (5 μmol/L/20 μl i.d.)-induced plasma extravasation in wild-type mice pretreated with or without the PAF receptor antagonist WEB2086 (10 mg/kg i.v.) or ABT491 (10 mg/kg i.v.), and digitized images used for density value evaluations (upper panels). Quantitative analysis of the images in the upper panels (lower panel). Values represent means ± SD. One-way ANOVA with Bonferroni’s test: *p < 0.05, **p < 0.01 (n = 5). (**a**–**d**) Similar results were obtained from at least two independent experiments.

We found that the extent of rhodocytin-induced plasma extravasation was significantly lower in mast cell–deficient W/Wv mice than in control mice (Fig. 2a). Reconstitution of mast cell–deficient W/Wv mice with wild-type bone marrow–derived mast cells (BMMCs) restored rhodocytin-induced plasma extravasation (Fig. 2a). We confirmed that the numbers of platelets in the circulation and CLEC-2 expression levels on platelets in W/Wv mice were comparable to those in wild-type mice (Supplementary Fig. 2a,b), although W/Wv mice had reduced numbers of red blood cells, as previously reported^25^. We also confirmed that rhodocytin activated platelets derived from W/Wv mice, as well as those from wild-type mice, as judged by CD62P (P-selectin) expression^26^ (data not shown). The lack of complete abrogation of compound 48/80–induced plasma extravasation in mast cell–deficient W/Wv mice (Fig. 2a) could be attributed to residual mast cells (<1%) in the skin of adult W/Wv mice^27^.

We also found that i.d. injection of rhodocytin increased plasma histamine levels in mice (Fig. 2b). Consistent with this, pretreatment of mice with the histamine H1 receptor antagonist olopatadine^28^, but not the platelet activation factor (PAF) antagonist WEB2086^29^ or ABT491^30^, decreased rhodocytin-induced plasma extravasation in the skin (Fig. 2c,d). We confirmed that both of the PAF antagonists significantly inhibited PAF-mediated plasma extravasation (Fig. 2d), indicating that these chemicals were functional *in vivo*. The different effects of WEB2086 and ABT491 on PAF-mediated plasma extravasation (Fig. 2d) might be due to differences in their inhibition constants (K_i_) for PAF-mediated responses (WEB2086: 11.9 nM; ATB491: 0.6 nM). Together, these results suggest that rhodocytin-induced plasma extravasation in mouse skin requires mast cells and histamine, but not PAF.

### Mast cells do not express CLEC-2

**Figure 3:**
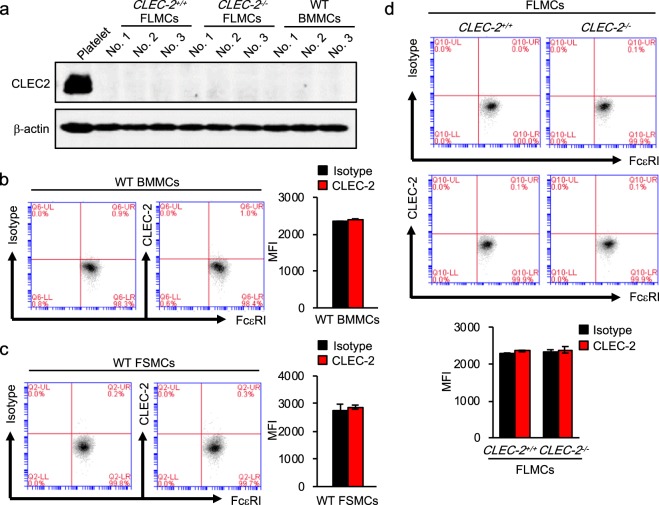
Mast cells do not express CLEC-2 at steady state. (**a**) Expression of CLEC-2 protein in wild-type BMMCs or FLMCs derived from CLEC-2^+/+^ or CLEC-2^−/−^ mice, detected by western blotting using anti–mouse CLEC-2 antibody (2A2B10) and anti–mouse β-actin antibody. Normal platelet lysate was used as a positive control for CLEC-2 detection. (**b**,**c**) CLEC-2 expression levels in BMMCs (**b**) or FSMCs (**c**) derived from wild-type mice, detected by flow cytometry (left panels), and quantitative analysis based on mean fluorescence intensity of Alexa Fluor 488–conjugated anti–mouse CLEC-2 antibody (right panels). (**d**) CLEC-2 expression levels in FLMCs derived from CLEC-2^+/+^ or CLEC-2^−/−^ mice, detected by flow cytometry (upper panels), and quantitative analysis based on mean fluorescence intensity of Alexa Fluor 488–conjugated anti–mouse CLEC-2 antibody (Lower panel). Values represent means ± SD. One-way ANOVA with Bonferroni’s test: *p < 0.05, **p < 0.01 (n = 5). Similar results were obtained from at least two independent experiments.

**Figure 4:**
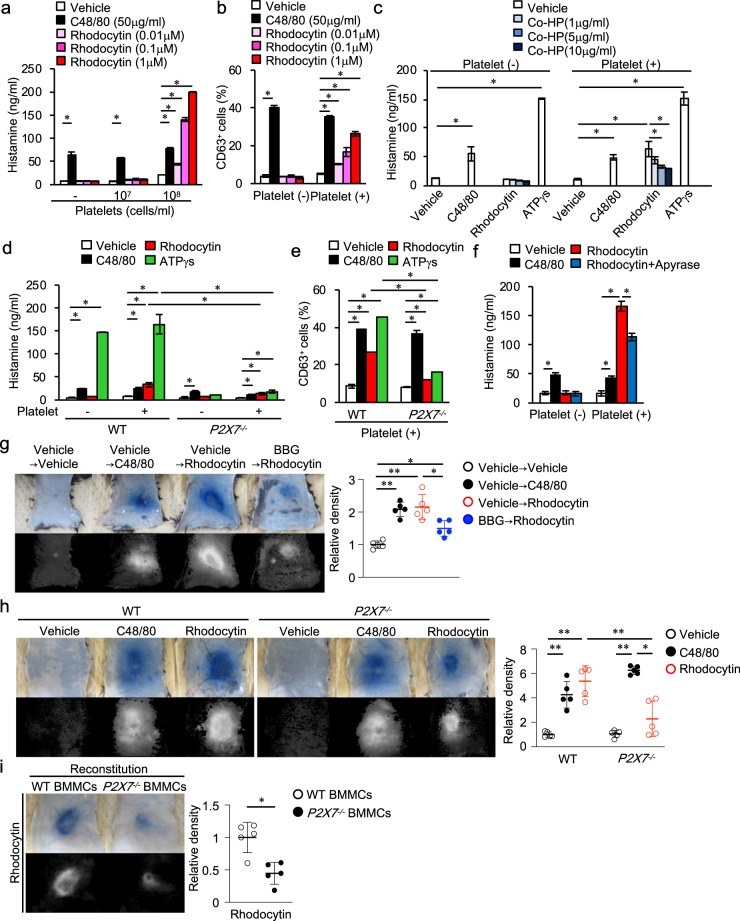
Rhodocytin/CLEC-2–mediated platelet activation induces histamine release from mast cells via the ATP/P2X7 pathway. BMMCs were co-cultured for 40 minutes in the presence or absence of C48/80, rhodocytin, or ATPγS, with or without mouse platelets. (**a**) Histamine concentration in supernatants of wild-type BMMCs co-cultured with the indicated numbers of mouse platelets and concentrations of rhodocytin. (**b**) CD63 expression on wild-type BMMCs co-cultured with or without mouse platelets (10^8^ cells/mL) in the presence or absence of C48/80 or rhodocytin. (**c**) Histamine concentration in the supernatants of wild-type BMMCs co-cultured with or without platelets (10^8^ cells/mL) in the presence or absence of C48/80 (50 μg/ml), rhodocytin (1 μmol/L), or ATPγS (200 μmol/L), with or without the indicated concentration of cobalt-hematoporphyrin (Co-HP). Co-HP was added to the co-culture 10 minutes before treatment with rhodocytin. (**d**) Histamine concentration in supernatants of wild-type or *P2X7*^−*/*−^ BMMCs co-cultured with or without mouse platelets (10^8^ cells/mL) in the presence or absence of C48/80 (50 μg/ml), rhodocytin (1 μmol/L), or ATPγS (200 μmol/L). (**e**) CD63 expression on wild-type or *P2X7*^−*/*−^ BMMCs co-cultured with or without mouse platelets (10^8^ cells/mL) in the presence or absence of C48/80 (50 μg/ml), rhodocytin (1 μmol/L), or ATPγS (200 μmol/L). (**f**) Histamine concentration in the supernatants of wild-type BMMCs co-cultured with or without mouse platelets (10^8^ cells/mL) in the presence or absence of C48/80 (50 μg/ml) or rhodocytin (1 μmol/L), with or without apyrase (1 U/mL). Apyrase was added to the co-culture 10 minutes before treatment with rhodocytin. (**g**) Representative images of C48/80 (10 μg/20 μl i.d.)- or rhodocytin (5 μmol/L/20 μl i.d.)-induced plasma extravasation in wild-type mice pretreated with or without P2X7 antagonist Brilliant Blue G (BBG; 50 mg/kg i.p.) (color), and digitized images used for density value evaluations (black and white) (left panels). Quantitative analysis of the images in the left panels (right panel). (**h**) Representative images of C48/80 (10 μg/20 μl i.d.)- or rhodocytin (5 μmol/L/20 μl i.d.)-induced plasma extravasation in wild-type or *P2X7*^−*/*−^ mice, and digitized images used for density value evaluations (left and middle panels). Quantitative analysis of the data in the left and middle panels (right panel). (**i**) Representative images of rhodocytin (5 μmol/L/20 μl i.d.)-induced plasma extravasation in mast cell–deficient W/Wv mice reconstituted with subcutaneous injections of wild-type or *P2X7*^−*/*−^ BMMCs, and digitized images used for density value evaluations (left panels). Quantitative analysis of the images in the left panels (right panel). Values represent means ± SD. One-way ANOVA with Bonferroni’s test: *p < 0.05, **p < 0.01 (n = 5). (**a**–**i**) Similar results were obtained from at least two independent experiments.

To investigate the possibility that rhodocytin directly activates mast cells, we measured the levels of CLEC-2 protein in wild-type BMMCs, fetal skin–derived mast cells (FSMCs)^31^ from wild-type mice, and fetal liver–derived mast cells from CLEC-2^+/+^ mice and CLEC-2^−/−^ mice^13^; the latter served as a negative control (Fig. 3a–d). Western blot and flow cytometry revealed little expression of CLEC-2 in these mast cells. Moreover, rhodocytin did not induce histamine in wild-type BMMCs *in vitro* (Fig. 4a). Thus, taken together with the earlier findings (Fig. 1), these observations indicate that rhodocytin induces plasma extravasation in mouse skin via indirect activation of mast cells, which subsequently leads to histamine release dependent upon CLEC-2 expressed on platelets.

### Rhodocytin induces histamine release from mast cells, dependent upon CLEC-2–mediated platelet activation

To investigate the mechanistic link between platelets, mast cells, and histamine in the context of rhodocytin-induced plasma extravasation, we co-cultured mast cells and platelets in the presence or absence of rhodocytin. Specifically, we cultured wild-type BMMCs (1 × 10^6^ cells/mL) with or without mouse platelets (1 × 10^7^ or 1 × 10^8^ cells/mL) in the presence of 0, 0.01, 0.1, or 1 μM rhodocytin, and then measured histamine concentrations in the culture supernatants (Fig. 4a). Compound 48/80 was used as a positive control.

Histamine was not detected in supernatants from cultures of wild-type BMMCs or mouse platelets alone in the presence or absence of rhodocytin, nor was it detected in supernatants from co-cultures of wild-type BMMCs with mouse platelets in the absence of rhodocytin. By contrast, addition of rhodocytin (0.01, 0.1, or 1 μM) to co-cultures of wild-type BMMCs and mouse platelets (1 × 10^8^ cells/mL) significantly increased histamine levels in a dose-dependent manner. Consistent with these findings, expression of CD63, a marker of activated mast cells^32^, increased when wild-type BMMCs (1 × 10^6^ cells/mL) were co-cultured with mouse platelets (1 × 10^8^ cells/mL) in the presence, but not the absence, of rhodocytin (Fig. 4b). We confirmed that the mouse platelets (CD41^+^FcεRIα^−^ cells) in these co-cultures were activated, as reflected by CD62P (P-selectin) expression, upon addition of rhodocytin (data not shown). Notably, the extent of histamine release from mast cells varied among experiments (~30–200 ng/ml), possibly due to differences in the rate of platelet activation by rhodocytin.

Recently we used ELISA to screen 6,770 compounds for their ability to inhibit CLEC-2/podoplanin binding. The screen identified protoporphyrin IX (H2-PP) as the most potent inhibitor. We then modified the hematoporphyrin moiety of this compound to complex with cobalt (Co-HP), which dramatically increased inhibitory potency relative to H2-PP^33^. Co-HP competitively inhibits the rhodocytin/CLEC-2 interaction on platelets, and hematogenous metastasis of podoplanin-expressing B16F10 to the lung in mice (which depends on tumor cell–induced platelet aggregation via CLEC-2/podoplanin interaction) is inhibited by intravenous (i.v.) administration of Co-HP^33^. Importantly, addition of Co-HP inhibited histamine release in co-cultures of wild-type BMMCs and mouse platelets in the presence of rhodocytin (Fig. 4c). These results suggest that rhodocytin induces histamine release from mast cells, dependent upon CLEC-2–mediated platelet activation.

### Rhodocytin/CLEC-2–mediated platelet activation induces histamine release from mast cells via the ATP/P2X7 pathway

Next, we sought to determine how rhodocytin induces histamine release from mast cells following CLEC-2–mediated platelet activation. We hypothesized that rhodocytin-activated platelets release one or more soluble factors that stimulate mast cells to release histamine. Hence, we screened several soluble factors released from rhodocytin-activated platelets upon histamine release from mast cells, and identified ATP as candidate soluble factor. Activated platelets release ATP^34^, and mast cells release histamine upon ATP stimulation via P2X7 receptor^35^. Indeed, we confirmed that ATPγS, which is hydrolyzed very slowly by most ATPases, induced histamine release from BMMCs derived from wild-type mice, but not P2X7-defieient mice^36^, regardless of the presence or absence of platelets (Fig. 4c,d).

Rhodocytin induced ATP release from platelets from wild-type mice (and also from *P2X7*-defieicent mice and W/Wv mice) *in vitro*, but this release was blocked by 10 μg/ml Co-HP (Supplementary Fig. 3a). Importantly, neither histamine nor CD63 expression was detected in supernatants from co-cultures of BMMCs derived from *P2X7*-deficient mice and mouse platelets in the presence of rhodocytin (Fig. 4d,e). Furthermore, the addition of apyrase, an ATP-degrading enzyme, to co-cultures of wild-type BMMCs with mouse platelets in the presence of rhodocytin significantly decreased histamine levels (Fig. 4f). We confirmed that *P2X7*-deficient BMMCs were comparable to wild-type BMMCs in terms of morphology and expression of mast cell markers (FcεRIα, CD117, mMCP-5, mMCP-6) (Supplementary Fig. 3b–d). We also confirmed that mouse platelets (CD41^+^FcεRIα^−^ cells) were activated upon addition of rhodocytin to the co-cultures, and that ATPγS did not directly activate mouse platelets, as judged by CD62P expression (data not shown).

Consistent with the *in vitro* findings, mice pretreated with the P2X7 antagonist Brilliant Blue G (BBG)^37^ and *P2X7*-deficient mice both exhibited reduced rhodocytin-induced plasma extravasation in comparison with vehicle-treated and wild-type mice, respectively (Fig. 4g,h). Importantly, reconstitution of mast cell–deficient W/Wv mice with BMMCs derived from *P2X7*-deficient mice, but not from wild-type mice, did not fully restore rhodocytin-induced plasma extravasation (Fig. 4i). We confirmed that the number of mast cells in W/Wv mice reconstituted with *P2X7*-deficient BMMCs was comparable to that in W/Wv mice reconstituted with wild-type BMMCs, as judged by toluidine blue staining (Supplementary Fig. 3e). We also confirmed that the numbers of platelets in the circulation and CLEC-2 expression levels on platelets in *P2X7*-deficient mice were comparable to those in wild-type mice (Supplementary Fig. 3f,g). Together, these *in vitro* and *in vivo* findings suggest that rhodocytin induces plasma extravasation by triggering platelet activation via CLEC-2, followed by activation of mast cells and histamine release via the ATP/P2X7 pathway.

### Significant numbers of platelets are located outside of blood vessels in the skin after vehicle or rhodocytin injections

**Figure 5:**
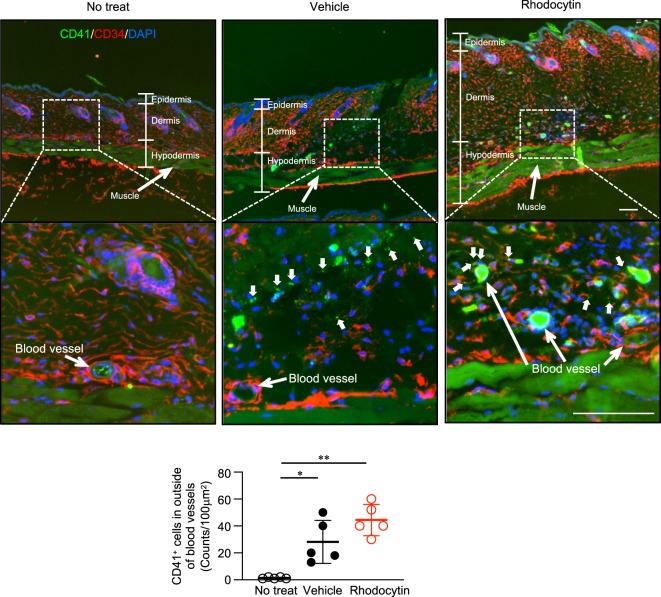
Significant numbers of platelets are located outside of blood vessels in the skin after vehicle or rhodocytin injection. Representative immunohistochemical staining images of dorsal skin with or without injections of vehicle or rhodocytin (5 μmol/L/20 μl i.d.). Dorsal skin was stained with antibodies against CD41 (platelets) and CD34 (blood vessels), as well as DAPI (nucleus). In vehicle- or rhodocytin-injected mouse skin, CD41^+^ platelets were observed outside of blood vessels as indicated by the bold white arrows (upper panels). Quantitative analysis of the images in the top panels (lower panel). Values represent means ± SD. One-way ANOVA with Bonferroni’s test: *p < 0.01, **p < 0.01 (n = 5). Scale bars, 100 μm. Similar results were obtained from at least two independent experiments.

Although the results above showed that platelet activation and subsequent ATP release by rhodocytin is required for plasma extravasation, it remained unclear how i.d. rhodocytin reached the platelets. Hence, we observed the sites of the i.d. injections of rhodocytin by immunohistochemical staining (Fig. 5). Intradermal injections physically damaged the injection site of the skin tissue regardless of the presence or absence of rhodocytin, and significant numbers of platelets were localized outside of blood vessels in skin tissue following the injections. This implied that i.d. injections might generate a microenvironment in which i.d. rhodocytin can reach platelets, and platelets can be close to mast cells in skin tissue.

### A chemical compound that competes with the rhodocytin/CLEC-2 interaction on platelets inhibits rhodocytin-induced plasma extravasation in mouse skin and hypothermia

**Figure 6:**
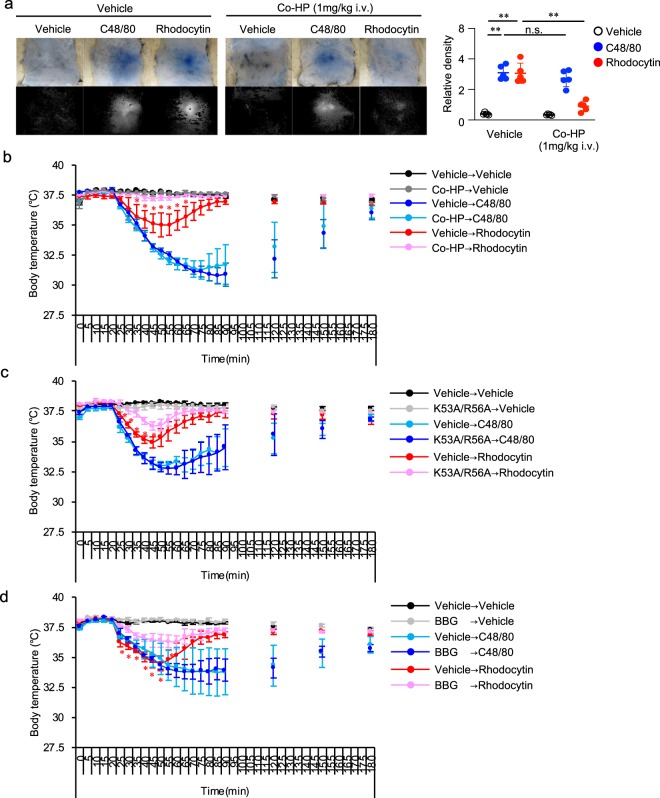
Cobalt-hematoporphyrin (Co-HP) inhibits rhodocytin-induced plasma extravasation and hypothermia in mice. (**a**) Representative images of C48/80 (10 μg/20 μl i.d.)- or rhodocytin (5 μmol/L/20 μl i.d.)-induced plasma extravasation in wild-type mice pretreated with or without cobalt-hematoporphyrin (Co-HP; 1 mg/kg i.v.) (color), and digitized images used for density value evaluations (black and white) (left panels). Quantitative analysis of the images in the left panels (right panel). Values represent means ± SD. One-way ANOVA with Bonferroni’s test: *p < 0.05, **p < 0.01 (n = 5). (**b**) Change in rectal temperature over time after i.v. administration of compound 48/80 or rhodocytin in wild-type mice treated with or without Co-HP (1 mg/kg). Co-HP was administered by i.v. injection 1 hour before administration of C48/80 (1 μg/kg) or rhodocytin (10 nmol/kg). (**c**,**d**) Change in rectal temperature over time after i.v. administration of rhodocytin in wild-type mice treated with or without K53A/R56A [20 nmol/kg i.v.] (**c**) or BBG [50 mg/kg i.p.] (**d**). K53A/R56A or BBG was administered by i.v. or i.p. injection 1 hour before administration of rhodocytin. Values represent means ± SD. One-way ANOVA with Bonferroni’s test: *p < 0.05 (n = 5). Similar results were obtained from at least two independent experiments. (**a**–**d**) Similar results were obtained from at least two independent experiments.

Next, we investigated whether Co-HP, a newly established chemical compound that blocks rhodocytin/CLEC-2 interaction on platelets^33^, could prevent rhodocytin-induced plasma extravasation. As expected, i.v. injection of Co-HP before i.d. injection of rhodocytin significantly suppressed rhodocytin-induced plasma extravasation in mouse skin (Fig. 6a). By contrast, i.v. injection of Co-HP did not affect the IgE/mast cell–mediated plasma extravasation (passive cutaneous anaphylactic [PCA] reaction) in mouse skin (Supplementary Fig. 4).

Systemic activation of mast cells and massive histamine release can induce peripheral vasodilation in many tissues, resulting in increased heat loss and hypothermia, which mimics a feature of hypovolemic shock. We found that systemic i.v. injection of rhodocytin induced hypothermia in wild-type mice. Consistent with the findings regarding plasma extravasation, Co-HP suppressed rhodocytin-induced hypothermia (Fig. 6b). Rhodocytin-induced hypothermia in wild-type mice was also suppressed by αWTβK53A/R56A (K53A/R56A)^21^ or the P2X7 receptor antagonist BBG^37^ (Fig. 6c,d).

Together, these findings suggest that chemical compounds that competitively inhibit the rhodocytin/CLEC-2 interaction on platelets, as well as P2X7 receptor antagonists, may be useful for preventing rhodocytin-induced plasma extravasation and hypothermia.

### Convulxin, a toxin from *Crotalus durissus terrificus* venom, does not induce plasma extravasation in the skin

Finally, we investigated whether other stimuli inducing platelet activation had similar effects as rhodocytin. For this purpose, we examined the effects of i.d. injections of the GPVI-activating toxin convulxin from *Crotalus durissus terrificus*^38^ on plasma extravasation in the mouse skin.

**Figure 7:**
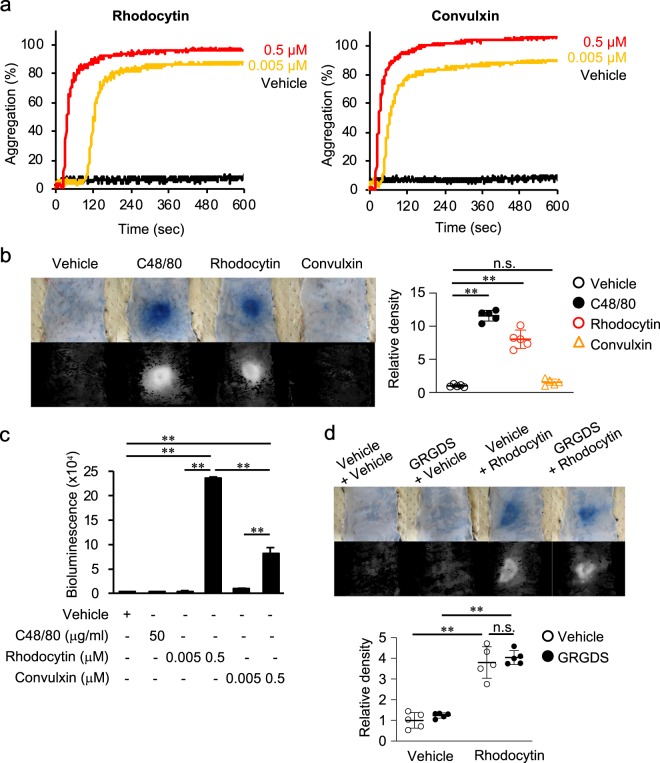
Convulxin, a toxin from *Crotalus durissus terrificus* venom, does not induce plasma extravasation in the skin. (**a**) Platelet aggregation was measured turbidometrically using an aggregometer. A 100-μl aliquot of washed platelets was incubated with rhodocytin or convulxin at the indicated concentrations in a cuvette in the aggregometer at 37 °C under constant stirring. (**b**) Representative images of C48/80 (10 μg/20 μl)-induced, rhodocytin (5 μM/L/20 μl)-induced or convulxin (5 μM/L/20 μl)-induced plasma extravasation in wild-type mice (color), and digitized images used for density value evaluations (black and white) (left panels). Quantitative analysis of the images in the left panel (right panel) (n = 5). (**c**) ATP release from platelets derived from wild-type (WT) mice in the presence or absence of C48/80, rhodocytin, or convulxin, as determined using an ATP bioluminescence assay. (**d**) Representative images of rhodocytin (5 μmol/L/20 μl)-induced plasma extravasation with or without the GPIIb/IIIa inhibitor GRGDS (1 μmol/L/20 μl) in wild-type mice (color), and digitized images used for density value evaluations (black and white) (left panels). Quantitative analysis of the images in the left panel (right panel). Values represent means ± SD. One-way ANOVA with Bonferroni’s test: *p < 0.05, **p < 0.01 (n = 5). N.S., nonsignificant difference. Similar results were obtained from at least two independent experiments.

We confirmed that convulxin induced platelet aggregation similarly to rhodocytin (Fig. 7a). Unexpectedly, however, convulxin did not induce plasma extravasation in the skin (Fig. 7b). Notably in this regard, convulxin induced significantly less ATP release from platelets than rhodocytin *in vitro* (Fig. 7c). These findings suggest that different venom toxins inducing platelet activation may have different effects on plasma extravasation in mouse skin and further support the notion that platelet ATP release, but not aggregation, is critical for plasma extravasation in mouse skin.

Consistent with these findings, GRGDS, an inhibitor of GPIIb/IIIa (RGD peptide) that suppresses platelet aggregation^39^, did not affect rhodocytin-induced plasma extravasation in the skin (Fig. 7d). Thus, induction of plasma extravasation by rhodocytin/CLEC-2 interaction depends on platelet activation and subsequent ATP release, and may be independent of platelet aggregation.

## Discussion

The precise mechanisms by which venomous snakebites induce plasma extravasation are not fully understood. The results of this study show that the venom rhodocytin from the Malayan viper *Calloselasma rhodostoma* induces plasma extravasation in the skin by triggering platelet activation via CLEC-2, followed by activation of mast cells and histamine release via the ATP/P2X7 pathway. The results reveal a previously unrecognized mechanism by which snake venom increases vascular permeability in the skin via complex venom toxin–mediated interactions between platelets and mast cells (Supplementary Fig. 5). This new pathway might play some roles in life-threatening pathophysiologies, such as hypovolemic shock or hypothermia induced by severe envenoming, along with non-specific tissue injury and inflammation. Additionally, our results reveal that platelets are key players in mast cell activation under particular conditions, and imply that platelets serve as sentinels for snake venom via their C-type lectin–like receptors.

Given that the rhodocytin/CLEC-2 interaction stimulates platelet activation and subsequent aggregation^4–6^, it is possible that rhodocytin induces plasma extravasation due to microthrombi and ischemia induced by platelet aggregation. However, at the dosages of rhodocytin used in these experiments, we did not observe any local or systemic signs of thrombosis or ischemia following i.d. injection of rhodocytin into mouse skin (data not shown). Importantly, GRGDS, a GPIIb/IIIa inhibitor that blocks platelet aggregation^39^, did not affect rhodocytin-induced plasma extravasation in the skin. Therefore, we assume that plasma extravasation induced by i.d. injection of rhodocytin is not a secondary effect of rhodocytin-mediated platelet aggregation and subsequent thrombosis or ischemia in the microenvironment.

The ATP/P2X7 pathway can activate mast cells, leading to histamine release^35^. P2X7 expressed on mast cells functions as a sensor for extracellular ATP, and is critical for maintaining the homeostasis of intestinal mucosa and skin^40,41^. ATP is stored in platelet granules at a concentration of ~200 mM, and is released upon activation^34^. We confirmed that rhodocytin induced ATP release from platelets derived from wild-type and *P2X7*^−*/*−^ mice *in vitro* in the absence of mast cells. Unfortunately, in our *in vitro* co-culture experiments, we were unable to detect ATP, probably because it is easily and rapidly degraded by ecto-nucleotide pyrophosphatase-phosphodiesterase 3 (E-NPP3) expressed on mast cells^42^. However, the observation that apyrase treatment or *P2X7* deficiency on mast cells decreased histamine release from mast cells supports the idea that rhodocytin can release extracellular ATP from platelets at sufficient concentrations for P2X7-mediated activation of mast cells.

Previous studies suggested that mast cells can be activated by the whole venoms of reptiles such as the Israeli mole viper or Gila monster^43–46^. In those cases, mast cells are likely to be activated directly by venoms containing peptides that are structurally similar to endogenous peptides, and are therefore recognized by innate receptors on the mast cell surface^46^. However, in this study, we found that rhodocytin did not directly stimulate mast cells, and mast cells did not express CLEC-2 at steady state. Thus, our findings provide another mechanism for mast cell activation by snake venom: some toxins may activate mast cells indirectly, with platelets or other cells acting as mediators.

Increased local vascular permeability/plasma extravasation is a major aspect of inflammation that contributes to host defense against pathogens by recruiting circulating leukocytes or increasing the concentrations of effector molecules in the extracellular space. In the case of snake or bee venoms, increased local vascular permeability may elevate interstitial concentrations of circulating inhibitors of toxic venom components, thereby enhancing innate venom resistance^46^. It is also known that snake or bee venom-induced mast cell degranulation contributes to innate venom resistance through the release of many kinds of proteases for degradation of the venom toxins^46^. Hence, we speculate that platelets activated via CLEC-2 promote innate venom resistance by inducing mast cell degranulation and subsequent plasma extravasation. It is also possible that leukocyte recruitment following mast cell degranulation may be protective against innate venom resistance or infection at the sites of the snakebites. However, excessive plasma extravasation can sometimes lead to deleterious outcome such as hypovolemic shock.

The limitations of this study are as follows.

1. Histamine may not be the only factor responsible for rhodocytin-induced plasma extravasation in the skin. In support of this idea, olopatadine, an H1 receptor antagonist, partially inhibited rhodocytin-induced plasma extravasation in the skin.
2. Similarly, factors other than ATP may play a role in mast cell activation and histamine release. Apyrase partially inhibited histamine release from mast cells *in vitro*, and the P2Y7 antagonist BBG and P2Y7-deficiency partially suppressed rhodocytin-induced plasma extravasation in the skin, as well as hypothermia. Because CXCL4 can induce mast cell degranulation via CXCR3^47^, CXCL4 released from platelets may be a candidate molecule.
3. We cannot exclude the possibility that ATP derived from endothelial cells or other cells damaged by i.d. injections of rhodocytin may be also important for rhodocytin-induced plasma extravasation in the skin. Indeed, rhodocytin can activate endothelial cells^48^.
4. The overall clinical response to *C*. *rhodostoma* bites cannot be explained by the effects of rhodocytin alone, as *C*. *rhodostoma* venom contains several other toxins^49^.
5. We have not mentioned about the possibility that ATP activated other innate immune cells, such as neutrophils, macrophages and eosinophils in the skin. These cells can be activated by ATP via purinergic P2 receptor^50,51^.
6. We found that rhodocytin/CLEC-2 stimulation releases much more ATP from mouse platelets than convulxin/GPVI stimulation. Thus, stimulation of different receptors on platelets may functionally separate platelet aggregation and ATP release. This issue is beyond the scope of this study, but we hope to investigate it in the future.
7. Because this study performed only in a mouse model, the relevance of the current findings to humans remains to be determined.

In summary, we propose a specific cellular and molecular mechanism underlying the elevated vascular permeability triggered by snake venom toxin in the skin. This mechanism may play a role in life-threatening pathophysiologies such as hypovolemic shock and hypothermia in cases of severe envenoming. In addition, the results suggest novel roles for platelets as mast cell activators and CLEC-2 as a key receptor for the innate response (i.e., plasma extravasation) to rhodocytin.

## Methods

### Mice

Male 6–8-week-old C57BL/6 mice and mast cell-deficient WBB6F1-W/Wv mice were purchased from Japan SLC (Tokyo, Japan). CLEC-2-null (CLEC-2^−/−^) mice were generated as previously described^13^. P2X7 receptor–deficient mice (*P2X7*^−*/*−^ mice on the C57BL/6 background)^36^ were kindly provided by Dr. Hiroshi Enaida (Saga University, Japan) and Dr. Shoji Notomi (Kyushu University, Japan). Mice were bred and maintained under specific pathogen–free conditions. All animal experiments were approved by the Institutional Review Board of the University of Yamanashi and carried out according to institutional guidelines (the reference number is A29-49).

### Reagents

Recombinant wild-type rhodocytin and mutated rhodocytins in which alanine replaced Asp4 (D4) in the α-subunit (D4A) or Lys53 (K53)/Arg 56 (R56) in the β-subunit (K53A/R56A) were generated in our laboratory^21^. Compound 48/80^22^, olopatadine (histamine receptor H1 antagonist)^28^, ATPγS, Brilliant blue G (BBG)^37^, Apyrase, WEB2086^29^, and ABT491^30^ were purchased from Sigma-Aldrich (St. Louis, MO, USA). Platelet-activating factor (PAF) was purchased from Tocris BioScience (Bristol, UK). The monoclonal anti-CLEC2 antibody 2A2B10 was prepared by the Cell Engineering Corporation (Osaka, Japan) as previously described^52^. Purified native rhodocytin^21^ was kindly provided by Dr. Yongchol Shin (Kogakuin University, Tokyo, Japan). Fibrinogen-related peptide Gly-Arg-Gly-Asp-Ser (GRGDS)^39^ was purchased from Peptide Institute (Osaka, Japan). The CLEC-2 antagonist Co-HP was generated in our laboratory^33^.

### Rhodocytin-induced plasma extravasation

Following i.v. injection of 0.5% Evans blue dye, mice were intradermally administered compound 48/80 (10 μg/20 μl, 500 μg/kg), wild-type rhodocytin (5 μmol/L/20 μL, 6 μg/kg), mutant rhodocytins (5 μmol/L/20 μL, D4A; 6 μg/kg, 10 μmol/L/20 μL, K53A/R56A; 6 μg/kg), or PAF (10 μmol/L/20 μL, 5.2 μg/kg). Olopatadine (10 mg/kg p.o.), WEB2086 (10 mg/kg i.v.), ATB491 (10 mg/kg i.v.), BBG (50 mg/kg i.p.), or Co-HP (1 mg/kg i.v.) was administered 1 hour before i.d. administration of rhodocytin and PAF. GRGDS (1 mM) was mixed with rhodocytin (5 μmol/L/20 μL) and administered intradermally.

Vascular permeability was visualized 30 minutes later based on blue staining of the injection sites on the reverse side of the skin. Staining sites were digitized using a high-resolution color camera (IXY3; Canon, Tokyo, Japan), and the images were saved in Windows Photo Viewer as 8-bit color-scale JPEG files. The open source ImageJ software package ver. 1.43 (NIH, USA) was used for image analysis, as previously described^20^. Briefly, color-scale images exported from Windows Photo Viewer were converted to HSB (“hue/saturation/brightness”) stack-type images using the Image tool. The HSB stack images were split into hue, saturation, and brightness images, respectively. Using the threshold tool, the blue-stained areas were selected from the hue image. These images were then combined with the saturation image and the density values for the blue-stained areas, and measured using the Analyze tool.

### Platelet depletion in mice

Platelet depletion in mice was achieved by single i.v. administration of 4 μg/g rat anti–mouse glycoprotein Ibα (GPIbα) antibody (#R300; Emfret Analytics, Würzburg, Germany) or 4 μg/g control rat IgG (Molecular Innovations, Novi, MI, USA) as previously described^53^. After 4 days, platelet depletion was confirmed by flow cytometry using APC-conjugated anti–mouse CD41 antibody (eBioscience) and Alexa Fluor 488–conjugated anti–CLEC-2 antibody (2A2B10).

### Depletion of CLEC-2 on platelets in mice

Depletion of CLEC-2 on platelets was achieved by a single i.v. injection of 8 μg/g rat anti–CLEC-2 antibody (2A2B10) or 8 μg/g control rat IgG (Molecular Innovations) as previously described^52^. After 3 days, CLEC-2 levels on platelets were detected by flow cytometry using APC-conjugated anti–mouse CD41 antibody and Alexa Fluor 488–conjugated anti–CLEC-2 antibody (2A2B10).

### Hemogram analysis

Blood samples were collected from wild-type, W/Wv, or *P2X7*^−*/*−^ mice in EDTA-coated blood collection tubes. Platelets and red blood cell counts were quantified on a Sysmex XE-2100 (Sysmex Corp. Kobe, Japan).

### Subcutaneous reconstitution of mast cells

Mast cell–deficient WBB6F1-W/Wv mice (Japan SLC) were reconstituted with subcutaneous injections of BMMCs (1.5 × 10^6^/mouse) derived from wild-type or *P2X7*^−*/*−^ mice. Six weeks after reconstitution, the mice were used for experiments^53^.

### Preparation of bone marrow–derived mast cells (BMMCs), fetal skin–derived mast cells (FSMCs), and fetal liver–derived mast cells (FLMCs)

Bone marrow–derived mast cells (BMMCs) were generated from the femoral bone marrow cells of male mice as previously described^54^. Fetal skin–derived mast cells (FSMCs) were generated from fetal skin of wild-type mice as previously described^31^. Fetal liver–derived mast cells (FLMCs) were generated from fetal liver cells from CLEC-2^−/−^ or CLEC-2^+/+^ mouse^13^ embryos on embryonic day (E) 15.5. Briefly, the fetal liver was gently crushed through a fine mesh, and the resultant cells were cultured for 4–6 weeks in RPMI 1640 medium (Sigma-Aldrich) supplemented with 1 mmol/L sodium pyruvate (Gibco), penicillin–streptomycin (Gibco), non-essential amino acids (Gibco), 10% heat-inactivated FCS (Gibco), 100 µmol/L 2-mercaptoethanol (Kanto Chemical, Japan), 10 ng/mL of murine interleukin-3 (PeproTech), and 10 ng/mL of murine SCF (PeproTech). Mast cells were identified by flow cytometric detection cell-surface c-kit (CD117) and FcεRIα.

### Western blot

Western blotting was performed as previously described^20^. Briefly, platelets or BMMCs were lysed by direct addition of sample buffer (Bio-Rad, Hercules, CA, USA). Cell lysates were electrophoretically removed in 10% SDS polyacrylamide gel and transferred onto Bio-Trace PVDF membrane (Pall Corporation, Port Washington, NY, USA). The membrane was incubated with primary antibody and an appropriate secondary horseradish peroxidase–conjugated antibody. Signals were detected using ECL (GE Healthcare Bioscience, Bucks, United Kingdom). Immunoreactive bands were visualized with the Chemi Doc XRS-J imaging system (Bio-Rad Laboratories) and analyzed with Quantity One (Bio-Rad) to determine their relative intensities. Primary antibodies were anti-CLEC-2 antibody (2A2B10) and anti-β-actin antibody (Santa Cruz Biotechnology, Dallas, TX, USA).

### Co-culture experiments

BMMCs were centrifuged at 400 *g* for 5 minutes and re-suspended (1 × 10^6^ cells/mL) in modified Tyrode’s buffer. The BMMC suspension was transferred into 96-well round-bottom plates (100 μL/well). Immediately, washed platelets (1 × 10^7^ cells/well) were added to the BMMC suspensions and stimulated with vehicle, C48/80, or recombinant rhodocytin in the presence or absence of apyrase (1 U/mL). Five minutes after stimulation, the BMMC–platelet mixture was collected, and CD62P-positive platelets were counted by flow cytometry. Forty minutes after stimulation, the BMMC–platelet mixture was centrifuged at 900 *g* for 10 minutes, and the cells were collected to count CD63-positive BMMCs by flow cytometry. The supernatants were also collected, and the levels of histamine in culture supernatants were measured by histamine EIA.

### Detection of ATP released from platelets

Detection of ATP released from platelets were measured with the ATP bioluminescence assay kit CLS II (Roche, Mannheim, Germany) according to the manufacturers protocol with some modifications. Briefly, washed platelets of wild-type, W/Wv or *P2X7*^−*/*−^ mice were adjusted with CFT buffer containing Ca^2+^ to the cell density of 2 × 10^8^ cells/mL. After the cell suspension and luciferase-luciferin reagent were mixed at equal volume, C48/80, rhodocytin or convulxin solution were added to a tube. After 5 minutes of reaction at 37 °C in the dark, the bioluminescence in a tube was measured by luminometer Gene Light 55 (Microtec Co., Ltd., Japan). Co-HP was added 5 minutes before addition of C48/80 or rhodocytin solution to the platelets/luciferase-luciferin reagent mixture.

### Measurement of histamine levels

The levels of histamine in culture supernatants or mouse plasma were measured with the histamine EIA kit (Oxford Biomedical Research, Oxford, MI, USA).

### Quantitative real-time PCR (Q-PCR)

Total RNA was purified from BMMCs using RNeasy Plus Mini Kit (Qiagen). The RNA samples were converted into cDNA with ReverTra Ace® (TOYOBO). Quantitative real-time PCR analysis was performed using the StepOne™ real-time PCR system (Applied Biosystems, Foster City, CA, USA), using primers and probes for mouse *mMCP-5*, *mMCP-6*, and *GAPDH* (Applied Biosystems) as previously described^20^. All mRNA levels were normalized against the level of *GAPDH* in the same sample. Data are provided as relative expression levels.

### *In vivo* staining and quantification of mast cells

Mouse tissue samples of back skin were fixed and embedded in paraffin, ensuring a cross-sectional orientation, and 2-mm sections were cut. Slides of paraffinized sections were dewaxed, rehydrated, and stained metachromatically with 0.05% toluidine blue (pH 4.1). Mast cells were counted in 10 fields of 1 mm^2^ within the wound healing area of one section per mouse, and the results from six sections per group were expressed as means ± SD.

### Immunohistochemistry

After 30 minutes rhodocytin i.d. injection, the dorsal skins collected from rhodocytin i.d. injected mice. These dorsal skins were then fixed with 4% paraformaldehyde (PFA) for 24 hours. After washing with diluted water, the dorsal skins were cut into longitudinal or transverse pieces. The pieces were subsequently incubated in 30% sucrose for cryoprotection. Treated tissues were embedded in OCT (Tissue-Tek, Sakura, Japan) at −80 °C. Frozen sections were prepared as previously described with a little modification^19^. Frozen dorsal skin sections sliced at 10 μm were blocked with Tris buffered saline (TBS) with Tween 20 containing 3% bovine serum albumin. Anti-CD41 Ab (MWReg30, Abcam, Cambridge, UK) and anti-CD34 (EP373Y, Abcam, Cambridge, UK) were diluted with blocking reagent and incubated for 1 hour at room temperature. Alexa Fluor 488-conjugated goat anti-rat Ab (for CD41, 1/2000 dilution, Abcam, Cambridge, UK) and Alexa Fluor 647-conjugated donkey anti-rabbit Ab (for CD34, 1/2000 dilution, Abcam, Cambridge, UK)were used as secondary antibodies. 4,6 Diamidino-2-phenylindole (DAPI) was used to counterstain the nucleus. After inclusion with Mounting PermaFluor (Thermo Fisher, Waltham, MA), sections were observed using an inverted fluorescence microscope (BZ-X800, Keyence, Osaka, Japan). Platelets (CD41^+^ cells) in the outside of blood vessels were counted in 5 fields of 100 μm^2^ of one section per mouse, and the results from five sections per group were expressed as means ± SD.

### Rhodocytin-induced hypothermia

Hypothermia in mice was induced with compound 48/80 or rhodocytin as previously described^43^ with some modifications. Briefly, mice were intravenously injected with compound 48/80 (1 μg/kg) or recombinant rhodocytin (10 nmol/kg). BBG (50 mg/kg i.p.), mutated-type rhodocytin (K53A/R56A; 50 nmol/kg i.p.) or Co-HP (1 mg/kg i.v.) was administered 1 hour before administration of each compound. Rectal temperature was measured with a digital thermometer (Shibaura Electronics, Tokyo, Japan) every 5 minutes after i.v. administration of rhodocytin.

### Platelet aggregation assay

Platelet aggregation assay was performed as previously described^21^. Briefly, mouse washed platelets (2 × 10^8^ cells/mL) were stirred at 1400 rpm in small cuvettes at 37 °C for 1 min on a platelet aggregometer, MCM Hema Tracer 712 (MC Medi-cal, Tokyo, Japan). Light transmission of washed platelets after addition of vehicle (Tyrode’s buffer), rhodocytin or convulxin was monitored by the MCM Hema Tracer 712 at 37 °C for 10 minutes.

### Statistical analysis

For two-group comparisons, statistical analyses were performed using the unpaired Student’s *t-*test. For multi-group comparisons, we applied one-way ANOVA with post hoc Bonferroni’s multiple comparison test. A value of p < 0.05 was considered to be significant, unless otherwise indicated.

## Supplementary information

Supplementary Informations
