# Supplementary material for: Snake venom rhodocytin induces plasma extravasation via toxin-mediated interactions between platelets and mast cells
Source: Sci Rep. 2019 Nov 4;9:15958. doi: 10.1038/s41598-019-52449-2 (PMC6828706; doi:10.1038/s41598-019-52449-2)
Supplement: Supplementary file 1 — Supplementary Informations [file 41598_2019_52449_MOESM1_ESM.pdf]

Supplementary information

Snake venom rhodocytin induces plasma extravasation via toxin-mediated interactions between platelets and mast cells

Yuki Nakamura,[1,*] Tomoyuki Sasaki,[2] Chihiro Mochizuki,[3] Kayoko Ishimaru,[1] Schuichi Koizumi,[4] Hideyuki Shinmori[5], Katsue Suzuki-Inoue,[2] & Atsuhito Nakao[1,6,*]

[1]Department of Immunology, [2]Department of Clinical and Laboratory Medicine,
Faculty of Medicine, University of Yamanashi,
1110 Shimokato, Chuo, Yamanashi 409-3898, Japan
[3]Research Center for Gold Chemistry, Graduate School of Urban Environmental Sciences, Tokyo Metropolitan University, 1-1 Minami-osawa, Hachioji, Tokyo 192-0397, Japan
[4]Department of Pharmacology, Faculty of Medicine, University of Yamanashi,
1110 Shimokato, Chuo, Yamanashi 409-3898, Japan
[5]Synthetic Biology Group, Department of Biotechnology, Faculty of Life and Environmental Science, University of Yamanashi, 4-4-37
Takeda, Kofu 400-8510, Japan
[6]Atopy Research Center, Juntendo University School of Medicine, 2-1-1 Hongo, Bunkyo-ku, Tokyo, 113-8421, Japan
[*]Correspondence to: Yuki Nakamura and Atsuhito Nakao (E-mail: ynakamura@yamanashi.ac.jp (YN) and anakao@yamanashi.ac.jp (AN)

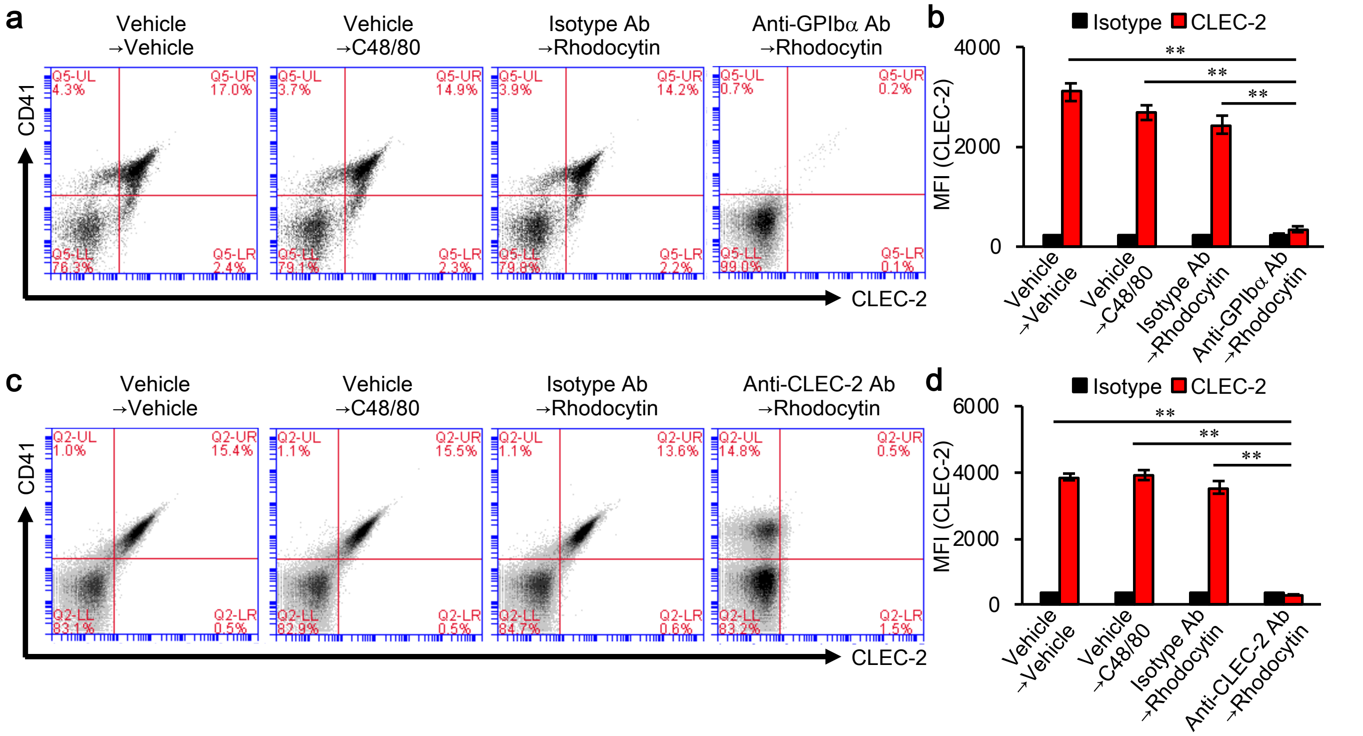

Supplementary Figure 1. Generation of platelet-depleted mice and platelet-selective CLEC-2–deficient mice
(**a**) Platelet depletion in mice was achieved by single intravenous administration of anti–mouse GPIba antibody or control IgG. After 3 days, the frequencies of $CD41^{+}CLEC\text{-}2^{+}$ platelets in mice were determined by flow cytometry.
(**b**) CLEC-2 expression levels on platelets were compared 3 days after injection of anti–mouse GPIba antibody or control IgG, based on the mean fluorescence intensity of Alexa Fluor 488–conjugated anti–mouse CLEC-2 antibody. (n = 5)
(**c**) Platelet-selective CLEC-2 depletion in mice was achieved by single intravenous administration of anti–mouse CLEC-2 antibody (2A2B10) or control IgG. After 4 days, the frequencies of $CD41^{+}CLEC\text{-}2^{+}$ platelets in mice were determined by flow cytometry.
(**d**) CLEC-2 expression levels in platelets were compared 4 days after injecting anti–mouse CLEC-2 antibody (2A2B10) or control IgG, based on mean fluorescence intensity of Alexa Fluor 488–conjugated anti–mouse CLEC-2 antibody. (n = 5)
One-way ANOVA with Bonferroni's test; values represent means ± SD, $^{*}p < 0.05$, $^{**}p < 0.01$.
Similar results (**a–d**) were obtained at least from 2 independent experiments.

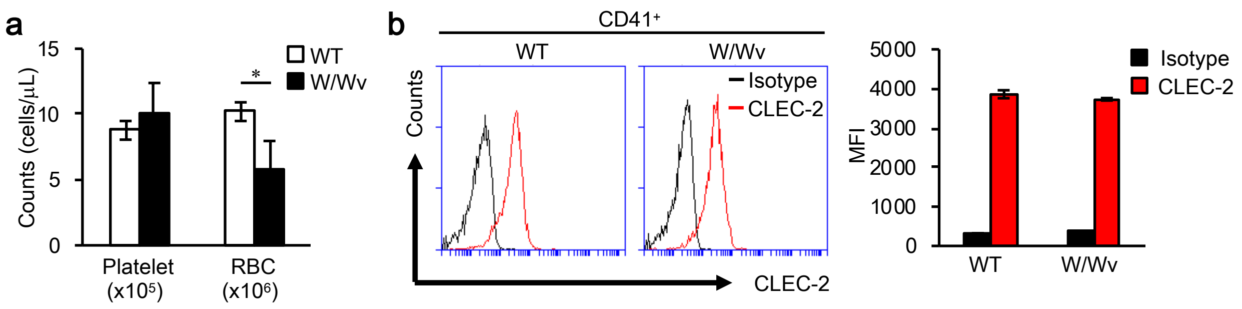

Supplementary Figure 2. Platelet levels and platelet CLEC-2 expression levels in W/Wv mice are comparable to those of wild-type mice

(**a**) Numbers of platelets and red blood cells (RBC) in W/Wv and wild-type mice, as determined by flow cytometry.

(**b**) Representative analysis of CLEC-2 expression levels in platelets ($CD41^+$) of W/Wv or wild-type mice, as determined by flow cytometry (**left panels**). CLEC-2 expression levels on platelets were compared, based on mean fluorescence intensity of Alexa Fluor 488–conjugated anti–mouse CLEC-2 antibody (**right panel**).

One-way ANOVA with Bonferroni's test; values represent means ± SD, $^*p < 0.05$, $^{**}p < 0.01$. (n = 5)

Similar results were obtained from at least two independent experiments.

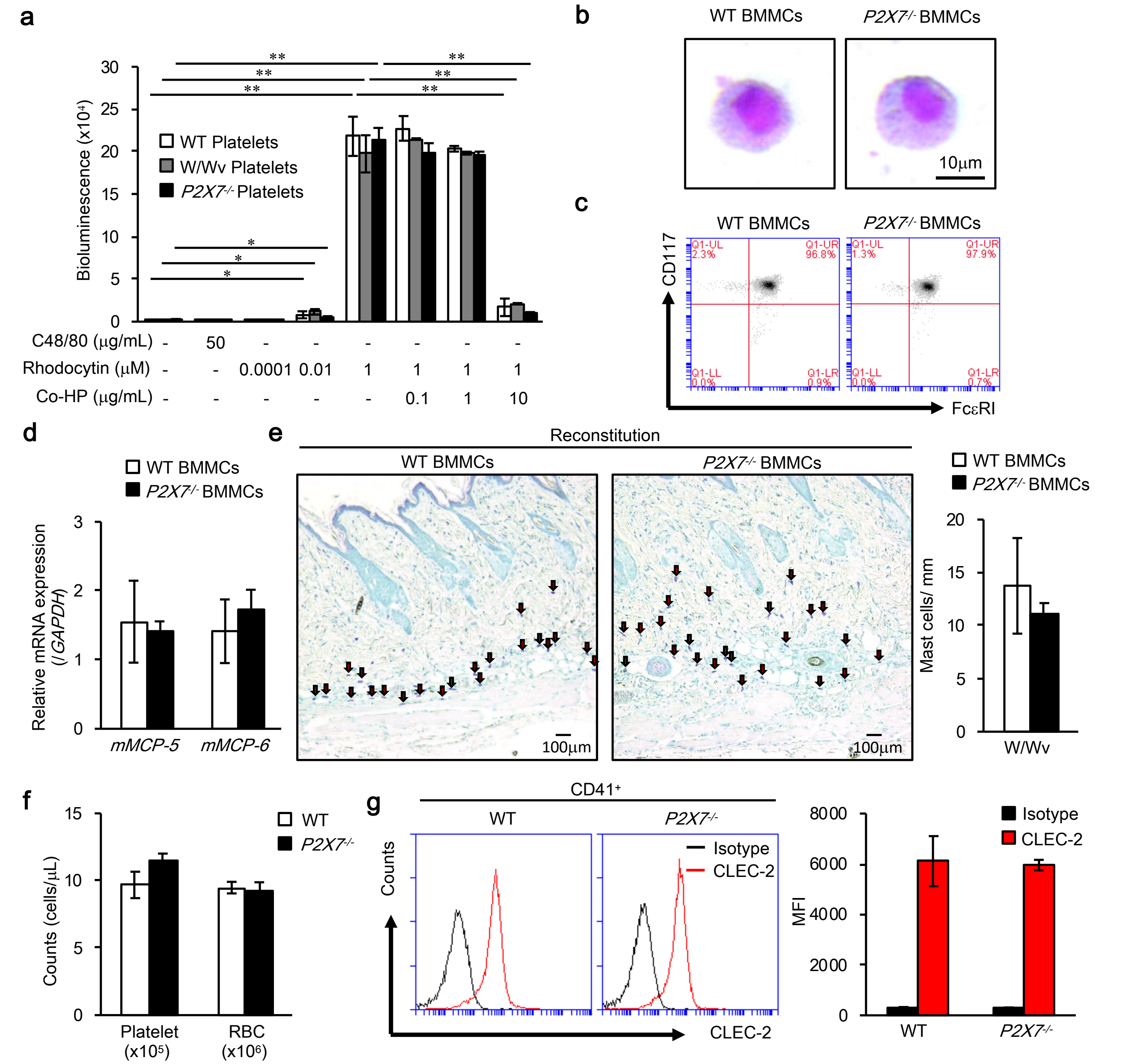

Supplementary Figure 3. Characterization of platelets and mast cells in *P2X7*-deficient mice

(**a**) ATP release from platelets derived from wild-type (WT), W/Wv, or *P2X7*-deficient (*P2X7*$^{-/-}$) mice in the presence or absence of C48/80 or rhodocytin, with or without Co-HP, as determined by ATP bioluminescence assay.

(**b–d**) BMMCs were generated from wild-type (WT) or *P2X7*-deficient (*P2X7*$^{-/-}$) mice. Five weeks after the start of bone marrow cell culture, Giemsa staining (**b**), flow cytometric analysis with anti-FcεRIa and anti–c-Kit (CD117) antibody (**c**), and real-time PCR analyses of *mMCP-5* and *mMCP-6* mRNA levels (**d**) were performed.

(**e**) Representative images of toluidine blue staining of mast cells (indicated by arrows) on the back skin of mast cell–deficient W/Wv mice reconstituted by subcutaneous injections (back skin) of BMMCs (1.5×10^6 per mouse) derived from wild-type (WT) or *P2X7*-deficient (*P2X7*$^{-/-}$) mice. Scale bar = 100 mm (**left panels**). Quantitative analysis of images in left panels (**right panel**) (n = 5).

(**f**) Numbers of platelets and red blood cells (RBC) in P2X7-deficine and wild-type mice, as determined by flow cytometry.

(**g**) Representative analysis of CLEC-2 expression levels in platelets of wild-type (WT) or *P2X7*-deficient (*P2X7*$^{-/-}$) mice, as determined by flow cytometry (**left panels**). CLEC-2 expression levels on platelets were compared based on mean fluorescence intensity of Alexa Fluor 488–conjugated anti–mouse CLEC-2 antibody (**right panel**).

Values represent means ± SD. One-way ANOVA with Bonferroni's test: *$p < 0.05$, **$p < 0.01$ (n = 5).

Similar results were obtained from at least two independent experiments.

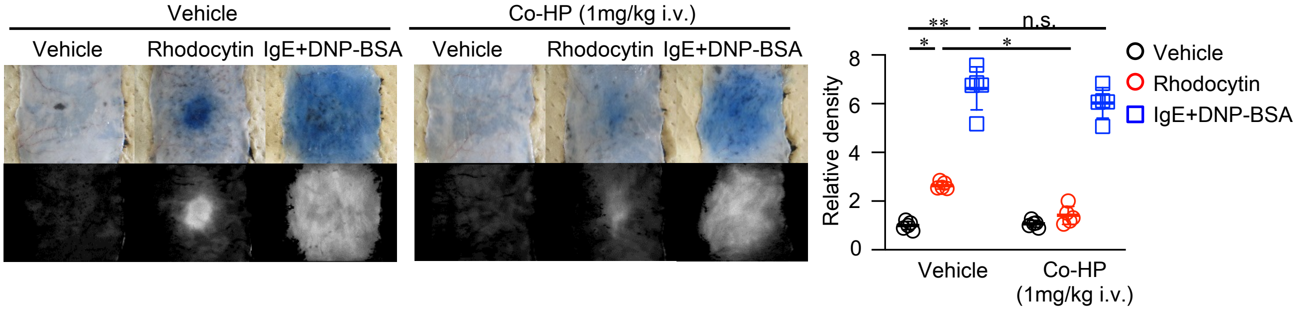

Supplementary Figure 4. Co-HP does not affect passive cutaneous anaphylactic (PCA) reaction in anti-TNP IgE–sensitized mice.

Representative images of rhodocytin-induced or DNP-BSA (DNP conjugated BSA)-induced plasma extravasation, with or without Co-HP (1 mg/kg i.v.), in wild-type mice (color), and digitized images used for density value evaluations (black and white) (**left panels**). Quantitative analysis of images in the left panels (**right panel**). Values represent means ± SD. One-way ANOVA with Bonferroni's test: $^{*}p < 0.05$, $^{**}p < 0.01$ ($n = 5$). Similar results were obtained from at least two independent experiments.

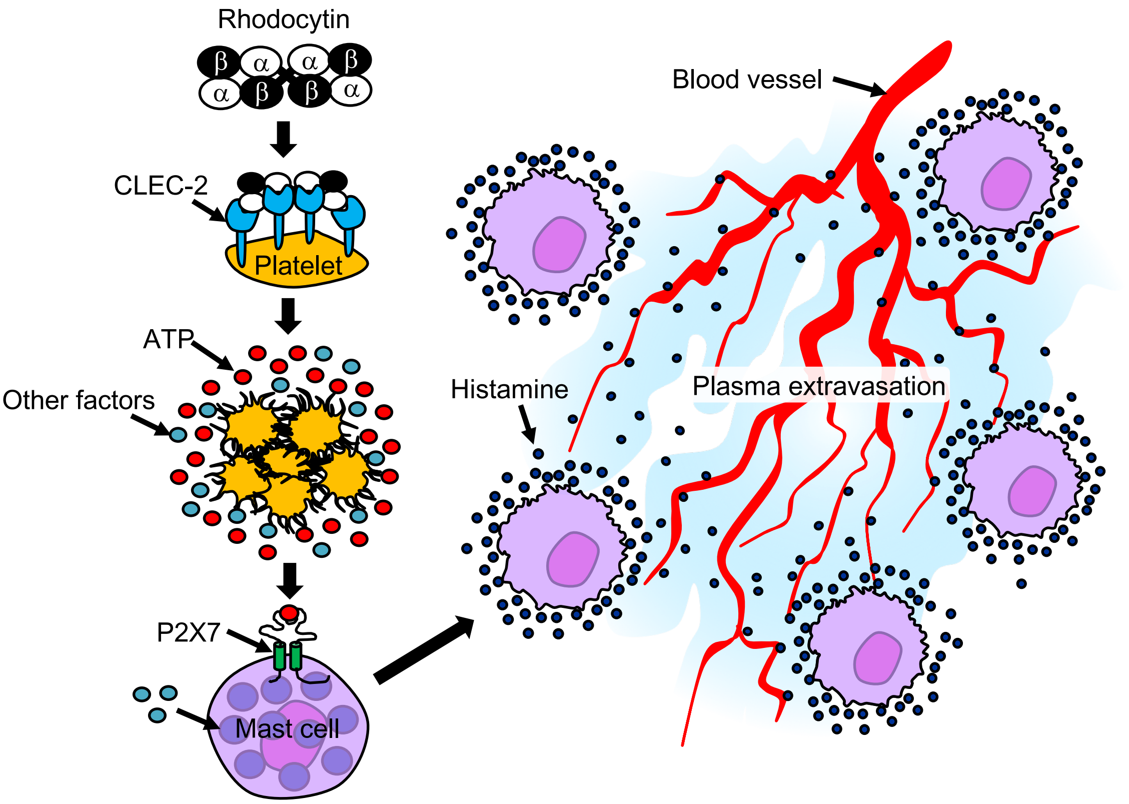

Supplementary Figure 5. Model

We propose that rhodocytin induces plasma extravasation by triggering platelet activation via CLEC-2, followed by activation of mast cells and histamine release via the ATP/P2X7 pathway. The results identify a previously unrecognized mechanism by which snake venom increases vascular permeability via complex venom toxin–mediated interactions between platelets and mast cells. This pathway may play a role in life-threatening pathophysiologies such as hypovolemic shock or hypothermia in cases of severe envenoming. Additionally, our results suggest novel roles for platelets as a mast cell activator and CLEC-2 as a key receptor for the innate response (plasma extravasation) to rhodocytin.

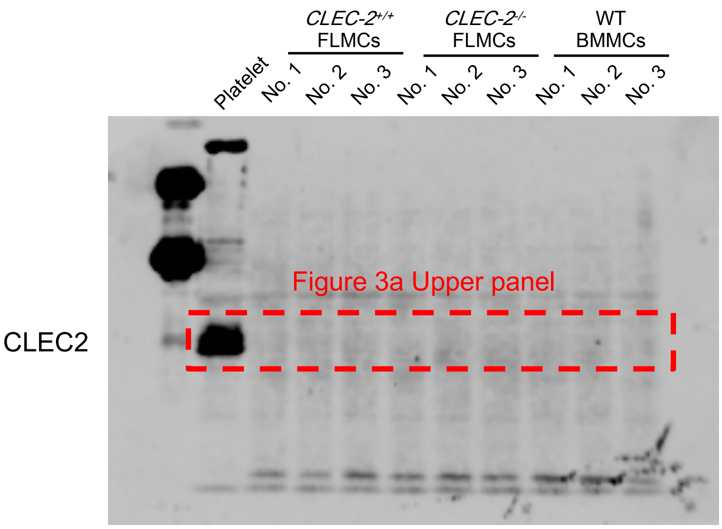

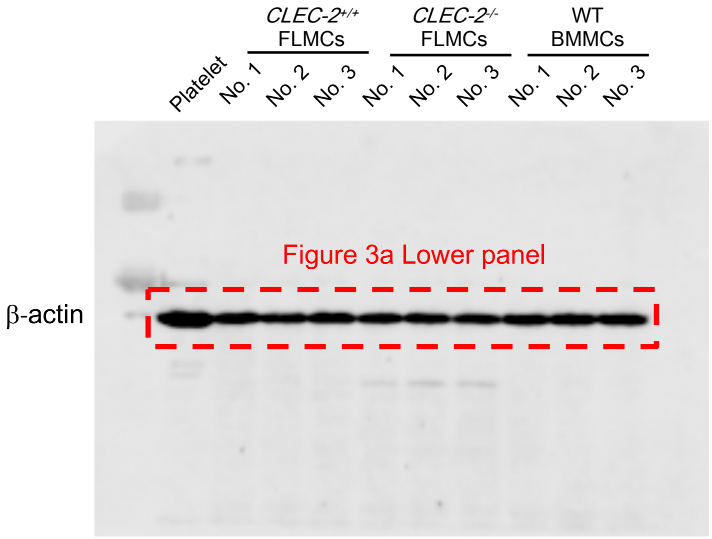

Supplementary Figure 6. Full unedited blot for Figure 3a
